# Visuomotor tracking strategies in children: associations with neurodevelopmental symptoms

**DOI:** 10.1007/s00221-023-06752-0

**Published:** 2023-12-11

**Authors:** Max Thorsson, Martyna A. Galazka, Mats Johnson, Jakob Åsberg Johnels, Nouchine Hadjikhani

**Affiliations:** 1 Gillberg Neuropsychiatry Centre, Institute of Neuroscience and Physiology, Sahlgrenska Academy, University of Gothenburg, Gothenburg, Sweden; 2 Division of Cognition and Communication, Department of Applied Information Technology, University of Gothenburg, Gothenburg, Sweden; 3 Section of Speech and Language Pathology, Institute of Neuroscience and Physiology, Sahlgrenska Academy, University of Gothenburg, Gothenburg, Sweden; 4 Athinoula A. Martinos Center for Biomedical Imaging, Harvard Medical School, Massachusetts General Hospital, Boston, MA USA

**Keywords:** Motor regulation, Visual motor skills, Neurodevelopmental disorders, Tablet-based testing, Error correction

## Abstract

Children with neurodevelopmental disorders (NDDs) often display motor problems that may impact their daily lives. Studying specific motor characteristics related to spatiotemporal control may inform us about the mechanisms underlying their challenges. Fifty-eight children with varying neurodevelopmental symptoms load (median age: 5.6 years, range: 2.7–12.5 years) performed an interactive tablet-based tracking task. By investigating digit touch errors relative to the target’s movement direction, we found that a load of neurodevelopmental symptoms was associated with reduced performance in the tracking of abrupt alternating directions (zigzag) and overshooting the target. In contrast, reduced performance in children without neurodevelopmental symptoms was associated with lagging behind the target. Neurodevelopmental symptom load was also associated with reduced flexibility in correcting for lateral deviations in smooth tracking (spiral). Our findings suggest that neurodevelopmental symptoms are associated with difficulties in motor regulation related to inhibitory control and reduced flexibility, impacting motor control in NDDs.

**Supplementary Information:**

The online version contains supplementary material available at 10.1007/s00221-023-06752-0.

## Introduction

A growing body of research provides evidence that neurodevelopmental disorders (NDDs) often have motor-associated atypicalities (Christiansen 2000; Kadesjö and Gillberg 1998; Yasumitsu-Lovell et al. 2021), suggesting that motor alterations are closely linked to other developmental symptoms (Bedford et al. 2016). Here, we address whether characteristics observed in children with neurodevelopmental symptoms are associated with a deficiency in functions responsible for the regulation and error correction in timed motor control.

Motor tasks in daily life range in complexity and demand. They can include basic reaching movements, precise inter-joint timing such as when doing handcrafts, or fast-paced movement in games, music, or sports, where external timing becomes critical (Braun Janzen et al. 2014; Shadmehr et al. 2010).

Importantly, efficient motor coordination relies on interacting and balancing various interconnected skills, such as general motor development, sensory responses, attention, and regulation (Adolph and Franchak 2017; Hadders-Algra 2018; Lohse et al. 2014). These areas mature during typical development, resulting in increasing motor proficiency until adulthood. However, the developmental trajectory of motor function may be altered in individuals with NDDs (Villagomez et al. 2019), negatively impacting social participation (Ohara et al. 2019; Rosenblum and Engel-Yeger 2014), social interactions, and general quality of life (Bremer and Cairney 2018; Rasmussen and Gillberg 2000). Because of the importance of motor control across the lifespan, it is critical to study motor control across childhood and into adolescence (MacDonald & McIntyre 2019).

### Neurodevelopmental disorders and symptoms

NDDs are present in approximately 10% of all school-aged children. Sometimes referred to under the umbrella term Early Symptomatic Syndromes Eliciting Neurodevelopmental Clinical Examinations [ESSENCE] (Gillberg 2010), they include, amongst others, attention-deficit/hyperactivity disorder (ADHD), autism spectrum disorder (ASD), and developmental coordination disorder (DCD). An overlap between these conditions is the rule rather than the exception (Gillberg 2010; Jensen and Girirajan 2017; Kadesjö and Gillberg 2001; Reiersen et al. 2007).

DCD specifically entails severe motor problems impacting the individual’s daily life and is often co-occurring with other NDD diagnoses. For instance, DCD and ADHD co-occur in at least 50% of children (Kadesjö and Gillberg 1998; Rasmussen and Gillberg 2000). DCD also co-occurs up to 70% with language disorders (Scabar et al. 2006) and is often present in individuals with ASD (Dewey et al. 2007; Green et al. 2009; Miller et al. 2021; Sumner et al. 2016). Early identification of the presence of any NDD, rather than focusing on any specific diagnosis, can facilitate earlier intervention and more positive outcomes for children who will potentially get a more definite diagnosis later in life (Gillberg 2010). Several neurodevelopmental symptoms may have common genetic aetiology (Pettersson et al. 2013) and vary over time (Allegrini et al. 2020, 2022). Dimensional analyses, rather than diagnostic comparison alone, can therefore provide a more comprehensive understanding of neurodevelopmental symptoms (Choi et al. 2020; Morris-Rosendahl and Crocq 2020). The study of motor problems across development may be of particular importance for understanding NDDs, as motor function is important for socio-emotional processes (Northoff et al. 2021) and interactions with others (Delafield-Butt and Trevarthen 2015).

### Motor prediction and error correction

Successful movement execution involves predicting and planning a motor response (Shadmehr et al. 2010), and models that depict motor control can be referred to as forward models (Jordan and Rumelhart 1992), where motor and sensory errors are prospectively corrected (Miall and Wolpert 1996).

In children following a typical developmental trajectory, evidence of action prediction can already be observed at 6 months of age (Gredebäck et al. 2018) and becomes more sophisticated with age (Kanakogi and Itakura 2011; Souto et al. 2020). This developmental trajectory may diverge in NDDs, wherein the efficient utilization of sensory input could be hindered (Buckingham et al. 2016; Król and Król, 2019; Wilmut and Wann 2008) resulting in temporal delays (Wilmut and Wann 2008).

Precise timing is crucial for motor control (Hore et al. 1996, 1991). Timing of motor control can be evaluated using tasks requiring visuospatial tracking, in which an individual has to synchronize their movement with an externally moving object (Hove et al. 2017; Szelag et al. 2004; Tirosh et al. 2006; Trommer et al. 1988; Whitall et al. 2008). Adult performance on these types of tasks (e.g., dragging a lever) reveals that when tracking another person’s movement, the follower typically overshoots—or moves ahead, rather than undershoots—or falls behind the target movement (Noy et al. 2011). This highlights the importance of a forward model in motor actions, together with self-monitoring and adaptive control required for the correction of motor error. Such predictive tracking seems deficient in children and adolescents with NDDs, who show a larger offset to the target movement (Dubey et al. 2023; Thorsson et al. 2023; Tirosh et al. 2006; van der Meulen et al. 1991). Tirosh et al. (2006) who studied manual tracking in children with and without NDDs further suggested that attention deficits may not be the sole cause of tracking difficulties in NDDs and that impairments in visuomotor tracking could be associated with reduced self-monitoring or adaptive control needed for correcting movement (Kurdziel et al. 2015; Mohamed et al. 2019; Shiels and Hawk 2010). Reduced self-monitoring and adaptive control, previously noted in individuals with NDDs (Kagerer et al. 2004; Kurdziel et al. 2015; Pomè et al. 2023; Shiels and Hawk 2010) strengthen the expectations for motor differences.

### Longitudinal and lateral control

Predictive control in visuomotor tracking can be decomposed into longitudinal and lateral adjustments. Longitudinal adjustments occur along the axis of the movement direction, while lateral adjustments take place perpendicular to it. The most prominent adjustments occur in the longitudinal axis (Gordon et al. 1994; Peternel and Babič, 2019; Saunders and Knill 2004) and become particularly important in visuomotor tracking of abrupt alternating directions (e.g., zigzag), as this type of task requires sudden movement inhibition, which is difficult for children with NDDs (Gaddis et al. 2015; Kenner et al. 2010; Macneil et al. 2011; Mostofsky et al. 2003; Shim et al. 2008; Swann and Greenhouse 2014; Valori et al. 2022). In addition, zigzag tracking also requires rapid wrist and hand rotations, and involves the smooth inhibition of specific muscles (Stinear and Byblow 2003), which can be affected in children with NDDs (Gillberg et al. 1983). At the same time, tasks with alternating movements require efficient motor synergies, where groups of muscles activate together to perform the task. Although the proficiency for alternating movement generally improves with age (Bessette et al. 2020; Gasser et al. 2010; Largo et al. 2001), individuals with NDDs may continue to face particular challenges with these types of tasks (Emanuele et al. 2021, 2022; Oliveira et al. 2006).

The lateral axis, where the direction is regulated through continuous subtle adjustments (e.g., tracking a spiral or a circle) is equally important (Sarlegna and Mutha 2015; Shabbott and Sainburg 2009; Shadmehr and Krakauer 2008). In typical children, directional control develops from an early age (Mathew and Cook 1990), and correction of lateral deviations improves through childhood (Mackrous and Proteau 2016). Manual tracking requiring this type of control is less urate in children with NDDs (Kalff et al. 2003; Slaats-Willemse et al. 2005). In one study Kalff et al. (2003) found that decreased accuracy during a cursor-controlled smooth tracking task was associated with ADHD symptomatology and may be related to reduced self-regulation. Another study measuring cursor movement in a stop-signal task that focuses on response inhibition, found a significant association between impulsivity as measured by the Conners’ Adult ADHD Questionnaire and distance from the target (Leontyev and Yamauchi 2019). However, specific mechanisms for deficits in directional control are still relatively understudied in children with NDDs. The present study aims to explore the potential impact of NDDs on motor regulation and adaptiveness for directional control in a manual tracking task.

### The present study

Fine-tuned regulation and prediction of the future motor response are essential for continuous motor regulation and trajectory correction (Wolpert et al. 1995). Our working hypothesis is that, from an early age, children with NDDs deviate in functions closely associated with continuous motor regulation and control (Gurevitz et al. 2014; Hatakenaka et al. 2015; Kanakogi and Itakura 2011; Koterba et al. 2014; Mackrous and Proteau 2016), resulting in reduced flexibility (Rommelse et al. 2007; Slaats-Willemse et al. 2005) and failure of error correction (Kagerer et al. 2004; Kurdziel et al. 2015; Pomè et al. 2023; Shiels and Hawk 2010). To test this idea and specify the nature of the difficulties, we investigated motor regulation and correction in a visuomotor tracking task in which online correction was an integral aspect in a group of children varying in neurodevelopmental symptom load.

To date, relatively few studies have examined visuomotor tracking in children with NDDs (Dubey et al. 2023; Janmohammadi et al. 2020; Thorsson et al. 2023; Tirosh et al. 2006; van der Meulen et al. 1991), and the majority did not include dynamic visual feedback, which we believe is essential for evaluating specific mechanisms for control, especially when testing children with NDDs (Liu and Breslin 2013; Yang et al. 2014).

Trajectory predictions are an integral part of motor control, and adults tend to overshoot rather than undershoot the tracked target (Noy et al. 2011). This process has also been explored using a Bayesian model, namely the Kalman filter (Kalman 1960; López-Moliner et al. 2019). The filter blends an internal motor simulation with sensory feedback to adjust their respective weights over time, resulting in more accurate state estimates (Miall and Wolpert 1996). We adopted the idea of a forward model with flexible weighting between the previous error and prediction as a theoretical framework for interpreting the results from our simplified model. To reduce the potential sudden directional errors from unknown directions, the path was made visible in our tracking experiment. The participants’ offset coordinates were also rotated relative to the target movement direction, allowing us to study the control of longitudinal and lateral offset to the target (Squeri et al. 2010). In this simple control system, there are two dimensions composing the gap to the target, which were assumed to be regulated by a predictive model.

When tracking sudden changes of direction, the motor regulation needs to be flexible to smoothly either reduce or increase velocity to minimize the distance to the target. In other words, the weighting between prediction, prior error, and the model itself, needs to be flexible. Here we considered the situation of tracking a trajectory of alternating (zigzag) and continuous (spiral) directions, to investigate if predictive flexibility and corrections were related to the magnitude of neurodevelopmental symptoms affecting different path types. Given previous research on inhibition problems (Mandich et al. 2002; Mostofsky et al. 2003; Valori et al. 2022), we hypothesized that for children with more NDD-related symptoms, the error would be more pronounced in tracking the zigzags, and reduced performance would be caused by overshooting, reflecting reduced flexibility in motor regulation. Another possibility is that the complexity of the trajectory would result in the children with NDDs performing slower movements.

We then considered the situation of tracking a trajectory of continuous direction (spiral) requiring quick corrections for lateral variability. The predictive model needs to account for unwanted variability in the movement, to prospectively correct for lateral deviations. Based on previously identified problems with self-awareness and error correction in NDDs (Kagerer et al. 2004; Kurdziel et al. 2015; Pomè et al. 2023; Shiels and Hawk 2010), we expected that affected children would be more negatively influenced by the unsteadiness.

We considered age as an essential variable throughout our analysis, because it relates to the development of functions necessary for temporal control (Gasser et al. 2010; Kanakogi and Itakura 2011; Largo et al. 2001; Mackrous and Proteau 2016; Rueckriegel et al. 2008; Souto et al. 2020; Thorsson et al. 2023).

Given the limited research studying the motor aspect of NDDs, finding specific features related to flexibility and error correction could identify mechanisms behind the problems experienced by children with neurodevelopmental symptoms in daily life, improve awareness of these specific limitations and open possibilities to provide more appropriate support.

## Research questions

Our research questions (RQs) were outlined as follows:

### RQ1: longitudinal error

Does the load of neurodevelopmental symptoms influence motor regulation to the target along the longitudinal axis of a trajectory of

[**A**] abrupt alternating directions (zigzag) and [**B**] continuous direction (spiral)?

### RQ2: performance and longitudinal regulation in zigzag tracking

[**A**] Does the load of neurodevelopmental symptoms influence the tracking performance in visuomotor tracking of alternating directions and

[**B**] Does the load of neurodevelopmental symptoms change how the longitudinal position affects the tracking performance?

### RQ3: performance and perpendicular adaptiveness in spiral tracking

[**A**] Does the load of neurodevelopmental symptoms influence the tracking performance in visuomotor tracking of continuous direction and

[**B**] Does the load of neurodevelopmental symptoms change the adjustment for lateral deviations during tracking performance?

## Methods

### Subjects

In this study, 78 parents and their children were recruited through the Child Neuropsychiatry Clinic, Queen Silvia’s Children’s Hospital, Sahlgrenska University Hospital, Gothenburg (*n* = 5), personal contacts at the research clinic (*n* = 4), and preschools and schools in the surrounding area (*n* = 69). The study was approved by the Swedish Ethical Review Authority and written consent from caregivers was received before data collection. To be included, participants needed to be aged between 2 and 13 years, without known motor disorder (except DCD), and without severe intellectual disability. We recruited children both from the Child Neuropsychiatry Clinic and from preschools and schools to cover a wide range of symptoms. Note that approximately 10% of children are estimated to have some kind of NDD (Gillberg 2010), but not all are seen in the clinic if the level of impairment is not alarming to the parents or the teachers.

### Tablet-based movement tracking

**Fig. 1:**
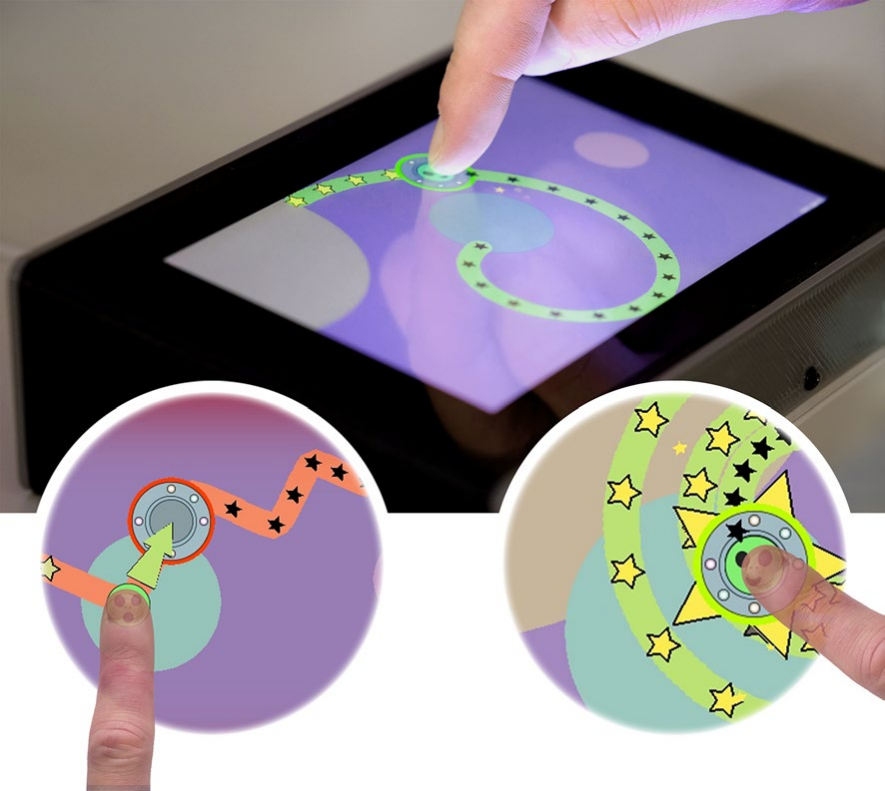
Upper panel: The tablet-based motor system presents an example trajectory, where the spaceship is being tracked by the fingertip. Left lower circle: Zigzag. Right lower circle: Spiral. Copyright© 2020–2023 Max Thorsson

Movement data were collected using an adaptation of the manual movement tracking task (*SpaceSwipe*) using a Raspberry Pi device that we previously found to be comparable to gold standard motor testing in children with a specific neurological condition [pediatric acute-onset neuropsychiatric syndrome, or PANS] (Thorsson et al. 2023). Here the game was adapted to suit younger children by adding more vivid colours and bigger stars, and by making the tracking paths more visible, so that the direction of the target movement would not be unexpected, see Fig. 1. Compared with Thorsson et al. (2023), here we specifically investigated mechanisms related to motor control, including error correction and predictive control, in relation to a dimensional measure of NDD symptom load and age.

The game starts with five easy levels before more complex trajectories are introduced, to ensure that the child understands the task. We focus on the final trajectories shown in the bottom panels of Fig. 1, which, to appear, require, that the child first successfully complete the five previous easier trajectories. Both the zigzag and spiral trajectories are of exponentially increasing curvature, and their speed profiles follow the power-law parameters for curvilinear hand movement suggested by Huh and Sejnowski (2015). Our variable for tracking alternating directions is based on the same zigzag path, in two different average speeds of 0.20 cm/s (4.5 s) and 0.43 cm/s (10.5 s). The variable for tracking of continuous direction is based on the same spiral path, tracked at two different average speeds of 0.20 cm/s (10.8 s) and 0.43 cm/s (24.5 s). The active game time, succeeding all levels on the first attempt is 1.4 min, and when including animations, is 3 min. Total testing time varies depending on the number of attempts by the participant to track individual trajectories.

**Fig. 2:**
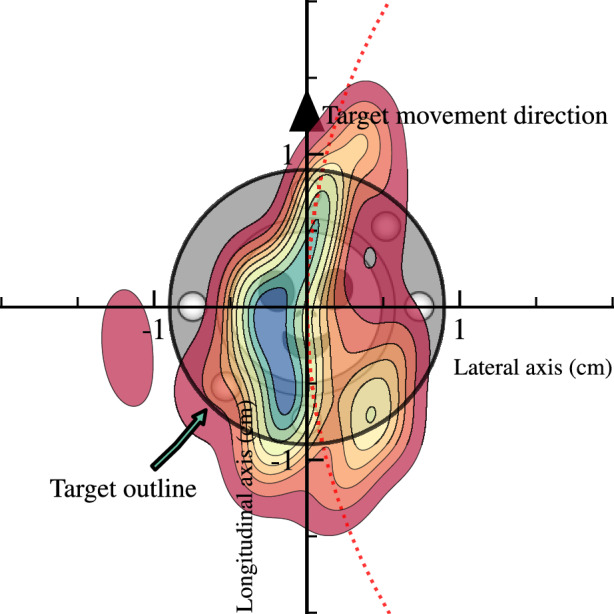
Illustration to display the resulting distribution of data points (kernel density estimation) of the rotated coordinates, centred on the middle of the target. The data points are from one participant tracking a trajectory. The dotted red line is not the actual trajectory but was added to display a hypothetical tangential angle

The objective of the tracking task is for the child to keep an alien inside a moving spaceship. The game includes visual feedback. Successful positioning within the spaceship is rewarded with a green glow around the spaceship and illuminated stars along the target path (see lower right in Fig. 1). If the touch is consistently > 7 mm away from the spaceship’s centre for 1.2 s, or if no touch is registered for 0.7 s, the level is reset. If the child is outside the spaceship area (see “Target outline” in Fig. 2), alerting feedback in the form of a red glow around the spaceship is initiated (see lower left in Fig. 1). This is done to motivate the child to follow the instructions. Each child is allowed to try multiple times. Trials in which the child completes at least 80%, are by design classified as successful and are included for analysis. This threshold was set before the data collection in the game logic and was consistent with the previous implementation (Thorsson et al. 2023).

To perform the task, each child was seated in a chair of proper height and was instructed how to play with the support of a task preview and visual game information cards similar to those used by Thorsson et al. (2023). To accommodate the wide age range, the game was designed to be of increasing difficulty, and it was therefore expected that younger participants would not reach the level of the final most complex trajectories.

### Data pre-processing

Touch screens can have internal noise. Therefore, the *x*- and *y*-touch screen coordinate time series are filtered using a zero-shift Butterworth low-pass filter, a common practice for filtering movement data (Bartlett 2007). We used a cut-off of 8 Hz with a 4th-order filter, following recent implementations (Chua et al. 2022; Thorsson et al. 2023). To investigate the effect of the path type, rather than their specific velocities, the metrics from the trajectories are standardized. This is done first per trajectory and then per type. To analyse the motor regulation, we create a projected space for longitudinal and lateral tracking control resulting in different metrics than the directional and spatial offset used in Thorsson et al. (2023). This is done by centring and rotating the participant's coordinates so that the direction of the target movement is the positive *y*-axis (the longitudinal axis, see Fig. 2). Time is not included, due to the constant capturing frame rate. First, the target movement’s tangential angle ($\phi$) with respect to the *y*-axis, is estimated using the 2-argument arctangent, where ${x^\prime},\,{y^\prime}$ are the *x*- and *y*-target tangential velocity, following (1),

1 $$\phi =atan2({x^\prime},\,{y^\prime}).$$

Finally, the rotated coordinates (${x}_{rot},\,{y}_{rot}$) are estimated (2),

2 $$\begin{aligned}{x}_{rot}=\left({x}_{participant}-{x}_{target}\right)\mathit{cos}\left(\phi \right)-\left({y}_{participant}-{y}_{target}\right)\mathit{sin}\left(\phi \right), \\{y}_{rot}=\left({x}_{participant}-{x}_{target}\right)\mathit{sin}\left(\phi \right)-\left({y}_{participant}-{y}_{target}\right)\mathit{cos}\left(\phi \right).\end{aligned}$$

### Main study variables

The following variables were used to examine motor regulation, variability, and task performance:

*Longitudinal error*—defined as the average longitudinal distance (in cm) between the participant's touch and the moving target’s position in the axis in the target movement direction (shown as the “Longitudinal axis” in Fig. 2). This metric captures the motor regulation error, as the extent of how much in front or behind, the participants were from the target position.

*Longitudinal position*—defined as the average position (in cm) relative to the moving target position on the axis of the target movement direction (shown as the “Longitudinal axis” in Fig. 2). This is used to capture relative over- or under-adjustment in the tracking regulation.

*Lateral variability*—defined as the standard deviation in the lateral position (in cm), on the axis perpendicular to the target movement direction (shown as the “Lateral axis” in Fig. 2). This metric is used for evaluating unsteadiness in directional control, representing movement direction adjacent to the target, therefore proportional to the angle between the touch position and the target direction. Both the longitudinal and the lateral variability are Euclidean distances in the transformed coordinate system.

*Tracking performance*—defined as the proportion of movement coordinates that are captured within the target area (see the circle annotated, “Target outline”, in Fig. 2). This evaluates the tracking performance, as the game objective was to keep the alien within the spaceship.

### ESSENCE-Q

The ESSENCE-Q was used to quantify the load of neurodevelopmental symptoms. The ESSENCE-Q is a one-page questionnaire completed by the parents, consisting of 12 items that assess various aspects of a child’s development, including general development, motor development, sensory reactions, communication, activity, attention, social interaction, behaviour, mood, sleeping, feeding, and “funny spells “/absences (Gillberg, 2012). The ESSENCE-Q have previously shown predictive validity for NDDs based on parental ratings in preschool (Cederlund 2022; Hatakenaka et al. 2017; Kattimani et al. 2022; Stevanovic et al. 2018) and school-aged children (Landgren et al. 2022). Each item offers response options of “Yes” (2 points), “Maybe/A little” (1 point), or “No” (0 points) for concern by the parents, or by someone else, for more than a few months. The total score ranges from 0 to 24 and represents the sum of “Yes” and “Maybe/A little” responses. Studies have identified different optimal cut-off scores for the presence of NDDs. Hatakenaka et al. (2017) found a score of 2 or more, Landgren et al. (2022), a score of 3 or more, and Kattimani et al. (2022), a score of 4 or more, as the most suitable cut-off for their samples. These were, however, different populations, with different administration of the questionnaire: Hatakenaka et al. (2017) examined children below 6 years, with the public health nurse or psychologist, filling out the forms. In, Landgren et al. (2022), children were 11 years old and parents filled out the forms. In Kattimani et al. (2022) pre-school children were examined with parents who filled in the forms. The use of the ESSENCE-Q score as a continuous variable in research is motivated by the fact that it directly asks for concerns for NDD symptoms in twelve domains, thereby giving information about the magnitude or load of NDD problems. The use of the total score as a continuous variable can provide more detailed information and statistical power (Altman & Royston 2006).

### Statistical analyses

The statistical analyses were performed using multivariate ordinary least squares (OLS), modelled with the statsmodels library (Seabold & Perktold 2010). Following the guidelines by Reid and Allum (2019) and implementations by other studies analysing motor-related features and development (Antunes et al. 2015; Bohannon et al. 2018; Hinton et al. 2007; Slining et al. 2010; Thorsson et al. 2023), and to account for the extent of performance improvement that may vary during development, both age and age-squared were included in the analysis. Interaction terms were used [RQ2B and RQ3B] to examine if neurodevelopmental symptoms influenced specific strategies for tracking performance. As the interaction terms increase multicollinearity, we mean-centred the concerned variables, following the recommendations by Iacobucci et al. (2017). A variance inflation factor (VIF) score above 5 typically indicates problematic multicollinearity (Kim 2019), and VIF scores were controlled for concerned variables in the regressions involving interaction terms. Robust covariance matrices were used in all regressions to reduce any potential effects of heteroscedasticity, as motivated by Hayes and Cai (2007). To get a continuous measure for the neurodevelopmental symptom load, we used the parent-filled ESSENCE-Q scores as a linear predictor of specific motor characteristics in visuomotor tracking.

## Results

Out of the 78 enrolled participants who were presented with the task, 4 (5.1%) children did not want to play, 4 (5.1%) did not finish the first game level, and 12 (15.4%) did not finish the final most complex trajectories. The children who did not perform the task and whose data were therefore not included in the analysis had a median age of 2.7 years (range: 2.1–3.6 years). The final sample consisted of 58 children (26 girls, 32 boys) of a wide age range (range: 2.7–12.5 years, median age: 5.7 years). Four of the 58 children (6.9%) only performed one of two trajectories of alternating directions and only trials that were completed were included in the analysis. Some participants tried but did not complete the level: slowest spiral (*n* = 3), the fastest spiral (*n* = 2), the slowest zigzag (*n* = 1) and the fastest zigzag (*n* = 3); these incomplete trials were not included. The total testing time for the sessions, including practice was in the median of 3.6 min.

Twenty-two (38.0%) of the children had 3 or more points in total ESSENCE-Q score, which had previously been identified as a screening cut-off for NDDs (Landgren et al. 2022). Using the higher cut-off of 4 or more points (Kattimani et al. 2022), 17 children (29.3%) were above the threshold. In total, 3 (5.2%) children had a clinical NDD diagnosis (1 ADHD + ASD, 1 ASD + ADHD + DCD, and 1 ADHD + dyslexia), 2 (3.4%) were under clinical investigation for NDDs, and 5 (8.6%) caregivers explicitly expressed that they suspected that the child had an NDD (3 ADHD, 2 language disorder, and 1 ASD).

**Fig. 3:**
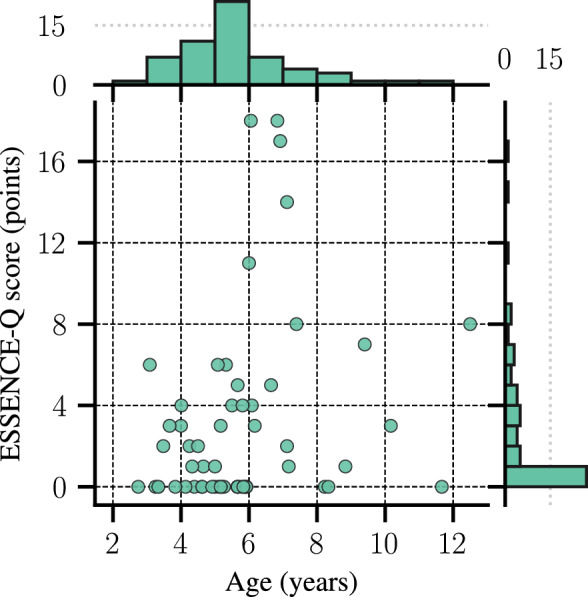
Scatterplot and histograms to display the distribution of age and ESSENCE-Q scores. The histograms (Upper: Age. Right: ESSENCE-Q) heights are the number of participants, *n* = 58

The ESSENCE-Q scores had a median of 1 and ranged from 0 to 18 points (*M* = 2.98, *SD* = 4.59). Figure 3 shows the distributions of age and ESSENCE-Q scores. The three most common items scoring positively were: activity (*n* = 9, 15.5%), attention (*n* = 8, 13.8%), and communication (*n* = 7, 12.1%); the most common scoring “Maybe/A little” were: attention (*n* = 10, 17.1%), mood (*n* = 10, 17.1%), and feeding (*n* = 9, 15.5%). We chose to use the entire questionnaire score instead of looking at separate item scores because they are strongly consistent with each other, as shown by a high Cronbach's alpha (*α* = 0.889, 95% CI [0.849, 0.922]). Furthermore, to ensure that the results were not affected by the question related to motor development, all results were re-evaluated excluding this item. This only resulted in minimal changes in decimals of coefficients and *p*-values, and therefore we decided to include the total scores, see the Supplementary material: Supplementary tables (without motor item). Details about the regressions and partial regression plots can be found in the Supplementary material.

### RQ1: longitudinal error

First, to investigate if the load of neurodevelopmental symptoms influenced the longitudinal error in the tracking of alternating directions (zigzag) [RQ1A], we employed a multiple linear regression to the longitudinal error (Longitudinal error ~ ESSENCE-Q + Age + Age^2^). We found that the ESSENCE-Q score exhibited a significant effect on the longitudinal error (*η*^2^ = 0.108, *β* = 0.095, *SE* = 0.037, *t*(56) = 2.588, *p* = 0.012). Age and age-squared were included as independent variables, to account for improvement in performance during development (see statistical analysis section), here and for all following regressions. Neither age nor age-squared showed a statistically significant relationship with the longitudinal error in the visuomotor tracking of alternating directions (*p*s ≥ 0.242).

Second, to investigate whether the load of neurodevelopmental symptoms influenced the longitudinal error in the tracking of *continuous direction* (spiral) [RQ1B], we employed a separate multiple linear regression to the longitudinal error in the tracking of continuous direction (Longitudinal error ~ ESSENCE-Q + Age + Age^2^). The ESSENCE-Q score did not show a significant effect on the longitudinal error (*p* = 0.132). By contrast, age showed a negative relationship with the longitudinal error (*η*^2^ = 0.179, *β* = −1.130, *SE* = 0.318, *t*(52) = − 3.555, *p* = 0.001), as did age-squared, with a smaller effect (*η*^2^ = 0.780, *β* = 0.050, *SE* = 0.021, *t*(52) = 2.345, *p* = 0.023). The significance and signs of the age terms imply that the error decreases with age, but that it may reduce less after a certain point.

In sum, our results indicate that a higher load of neurodevelopmental symptoms yields a larger longitudinal error in the tracking of alternating directions, whereas it is the age that is the main driver in the tracking of continuous direction. The non-significant relationship between neurodevelopmental symptom load and the longitudinal error in the tracking of continuous direction motivated us to further investigate the correction of lateral deviations [RQ3B].

### RQ2: tracking performance and longitudinal regulation in zigzag tracking

First, to investigate if the load of neurodevelopmental symptoms influenced the tracking performance in visuomotor tracking of *alternating directions* (zigzag) [RQ2A], we employed a separate multiple linear regression to the tracking performance, the standardized proportion of data points within the spaceship’s area (Tracking performance ~ ESSENCE-Q + Age + Age^2^). We found that the ESSENCE-Q score exhibited a significant effect on the tracking performance (*η*^2^ = 0.114, *β* = − 0.061, *SE* = 0.022, *t*(56) = − 2.754, *p* = 0.008). Tracking performance was positively correlated with age (*η*^2^ = 0.012, *β* = 0.551, *SE* = 0.265,* t*(56) = 2.078, *p* = 0.043), but not with age-squared (*p* = 0.383). Collectively, this implies that the performance improved with age but was hindered by neurodevelopmental symptoms.

Second, to investigate if the load of neurodevelopmental symptoms was related to longitudinal regulation that affected tracking performance [RQ2B], we employed a multiple linear regression that included an interaction effect between the ESSENCE-Q score and the longitudinal position. The longitudinal position showed a positive relationship with tracking performance (*η*^2^ = 0.010, *β* = 0.385, *SE* = 0.117, *t*(56) = − 3.295, *p* = 0.002), indicating that being further in the target movement direction related to how well the participants could keep their fingertips within the target area.

In contrast, the ESSENCE-Q score did not show a significant relationship to tracking performance (*p* = 0.123). Age still showed a positive relationship with tracking performance (*η*^2^ = 0.360, *β* = 0.718, *SE* = 0.251,* t*(56) = 2.861, *p* = 0.006), whereas age-squared did not (*p* = 0.102). Notably, the interaction term between longitudinal position and ESSENCE-Q score had a negative relationship with tracking performance (*η*^2^ = 0.072, *β* = − 0.040, *SE* = 0.013, *t*(56) = − 3.047, *p* = 0.004). VIF scores for the concerned variables were ≤ 1.878, indicating low multicollinearity and confidence in the reliability of the coefficient estimates for the predictor variables. This implies that the combination of being more behind the target and having a higher ESSENCE-Q score has a less clear association with poor tracking performance.

Furthermore, to ensure that the overshooting was not a result of the participants’ touch being in a sharp turn in the zigzag, causing erroneous interpretation of the longitudinal positioning, were evaluated the results in the zigzag segmented at sharp turns. As only minimal changes in decimals of coefficients and *p*-values, meaning that the interpretation was valid, see Supplementary material: Supplementary testing (zigzag).

**Fig. 4:**
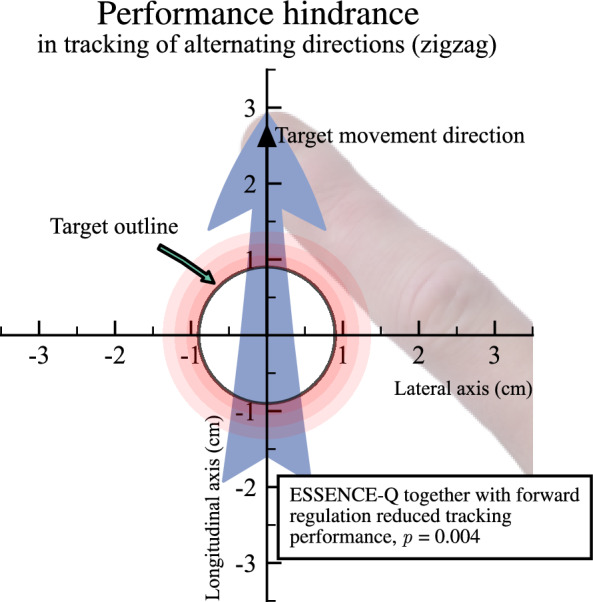
Graphical representation of how the over-adjustment (in the direction of the blue arrow) together with NDD symptom load reduced tracking performance

In sum, we found that age is an important predictor for tracking performance in visuomotor tracking of alternating directions. Moreover, we found that the relationship between longitudinal regulation and tracking performance varies depending on the load of neurodevelopmental symptoms. This suggests that children with more NDD-related issues perform worse by over-adjustments (i.e., being in front of the target), and benefit more from being slower in the tracking compared to the children with fewer or no NDD-related symptoms, who benefited from being faster. Figure 4 shows how forward regulation together with neurodevelopmental symptoms reduced tracking performance.

### RQ3: tracking performance and perpendicular adaptiveness in spiral tracking

First, to investigate if the load of neurodevelopmental symptoms influenced tracking performance in visuomotor tracking of continuous direction (spiral) [RQ3A], we employed a separate multiple linear regression to the tracking performance (Tracking performance ~ ESSENCE-Q + Age + Age^2^). We found that age had a positive relationship with tracking performance (*η*^2^ = 0.230, *η*^2^ = *β* = 1.252, *SE* = 0.295, *t*(52) = 4.243, *p* < 0.001) that decreased with age-squared (*η*^2^ = 0.111, *β* = − 0.058, *SE* = 0.020, *t*(52) = − 2.952, *p* = 0.005). This suggests that performance improves with age, but it may improve less after a certain point. In contrast, the ESSENCE-Q score did not show a significant relationship with tracking performance (*p* = 0.215).

Second, we investigated if the load of neurodevelopmental symptoms was related to the correction for lateral deviations [RQ3B], by including an interaction between lateral variability and the ESSENCE-Q score. In this model, we found a negative relationship between lateral variability and tracking performance (*η*^2^ = 0.211, *β* = − 0.676, *SE* = 0.074, *t*(52) = − 9.160, *p* < 0.001). This suggests that unsteadiness in the tracking of continuous direction reduced the tracking performance.

The ESSENCE-Q score demonstrated a non-significant negative relationship with tracking performance (*η*^2^ = 0.005, *β* = − 0.018, *SE* = 0.010, *t*(52) = − 1.732, *p* = 0.090) and age showed a non-significant positive relationship (*η*^2^ = 0.507, *β* = 0.382, *SE* = 0.198,* t*(52) = 1.925, *p* = 0.060). Age-squared was not significant (*p* = 0.239).

The interaction between lateral variability and ESSENCE-Q score had a negative relationship with tracking performance (*η*^2^ = 0.023, *β* = − 0.045, *SE* = 0.011, *t*(52) = − 4.014, *p* < 0.001). VIF scores for the variables of interest were ≤ 2.250, indicating low multicollinearity and confidence in the reliability of the coefficient estimates for the predictor variables.

Furthermore, to ensure that the lateral variability was not a result of the participants accurately tracking the spiral but with a delay relative to the target, causing an ambiguous interpretation of the variability, we evaluated the results by considering the closest position, rather than the target position, on the spiral path as a reference point. As only minimal changes in decimals of coefficients and *p*-values, meaning that the interpretation was valid, see Supplementary material: Supplementary testing (spiral).

**Fig. 5:**
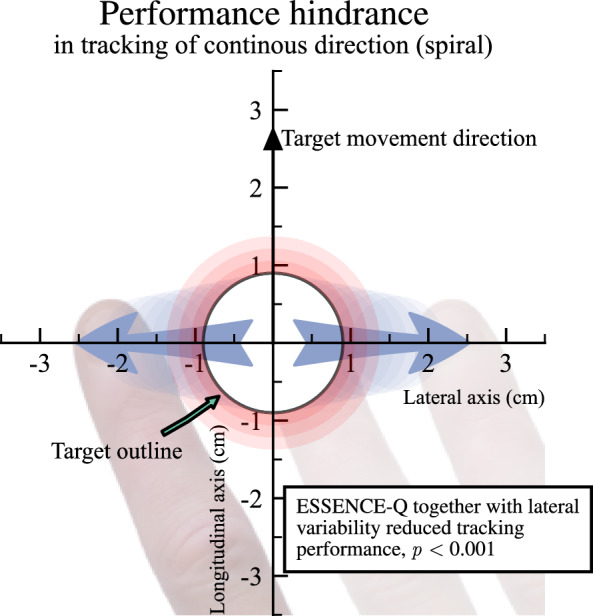
Graphical representation of how lateral variability (shown as green horizontal arrows) together with NDD symptom load reduced tracking performance

In sum, we found that older children have better tracking performance when tracking continuous direction and that less lateral variability leads to more successful tracking. Importantly, we found that increased neurodevelopmental symptom load negatively affects tracking performance and increases lateral variability. Figure 5 shows how lateral variability together with neurodevelopmental symptom load reduces tracking performance.

## Discussion

**Fig. 6:**
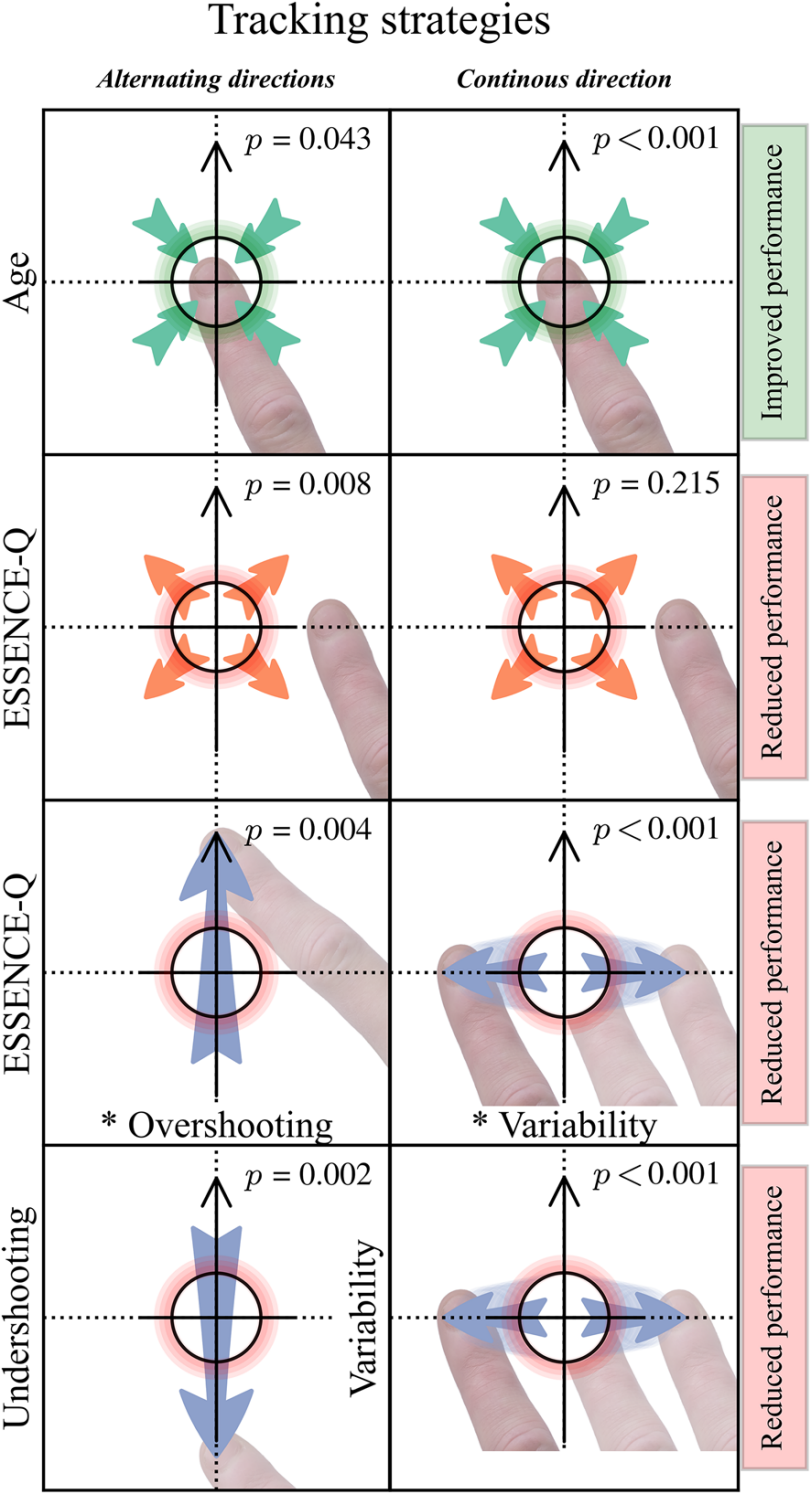
Illustration of our main results in tracking of alternating and continuous directions, in relation to ESSENCE-Q score and age. Predictors are shown on the *y*-axes and interaction terms marked, *. The effect on performance is shown to the right. The black circle represents the tracking target’s outline and the black upward arrow, the target movement direction. The blue arrows display the direction of the participants’ offsets. *p*-values are from *t*-tests, see more specific details in the results section

Our findings demonstrate that neurodevelopmental symptoms are associated with visuomotor function difficulties, in particular the tracking of alternating directions such as a zigzag. Impairments were specifically associated with longitudinal errors, in the form of over-adjustments. Performance, however, was less affected in the tracking of continuous direction (spiral), and children with more neurodevelopmental symptom load only showed a reduction in performance, observed with lateral variability. Neurodevelopmental symptoms limited the correction for lateral deviations, hence reducing tracking performance. Age showed an overall positive association with tracking performance. Our main results are summarized in a visual representation in Fig. 6.

Our findings of difficulties with visuomotor tracking in children with neurodevelopmental symptoms are in line with findings from several other prior studies (Dubey et al. 2023; Thorsson et al. 2023; Tirosh et al. 2006). There are, however, specific similarities and distinctions in relation to prior research that are worth discussing. To begin, we used a tracking task that gave interactive visual feedback throughout the motor execution, meaning that participants were notified if they were outside the target area, and the level was reset if they did not follow the target. This potentially made online error correction play a role in performance, as children were prompted to follow the task. Notably, Dubey et al. (2023) also included some type of visual feedback in their experiment in which participants were asked to track an insect on a touch-screen, and where previous coordinates were shown as a path, during the tracking—yet this information did not provide any feedback regarding accurate performance.

Our study also specifically investigated motor characteristics in regulation and error correction in visuomotor tracking which has, to our knowledge, not been done previously in relation to neurodevelopmental symptoms. Our findings on motor regulation may relate to a successful motor prediction in children without diagnoses, corroborated by the fact that a preference for overshooting in tracking has been found in adults without diagnoses (Noy et al. 2011). However, if children had neurodevelopmental symptoms, they showed a larger longitudinal error, and their predictive strategy negatively affected performance in the tracking of alternating trajectories. The relationship between a higher load of neurodevelopmental symptoms and a larger longitudinal error may be an indication of specific problems with motor regulation in timed movement requiring controlled alternations.

Dahan and Reiner (2017) argued that inhibitory problems may be induced by deficits in motor planning. Here, by having visible paths and giving visual feedback throughout the task, we tried to minimize requirements for directional planning before the movement initiation, underlining the importance of online correction. However, our findings do not rule out the role of continuous motor planning (Dahan & Reiner 2017; Valori et al. 2022), needed for continuous movement, as mentioned by Valori et al. (2022).

Current results corroborate previous findings on the relationship between spatiotemporal control and age evidenced in a very similar tracking task (Thorsson et al. 2023). This is not surprising, as functions responsible for spatiotemporal control are known to improve with age (Gasser et al. 2010; Kanakogi & Itakura 2011; Largo et al. 2001; Mackrous & Proteau 2016; Rueckriegel et al. 2008; Souto et al. 2020; Thorsson et al. 2023). The finding that the tracking performance improved with age for both continuous and alternating trajectories can be interpreted as an indication that the tasks capture features related to the development of motor functions for predictive control. It is, however, important to note that age may affect other aspects of the testing situation such as postural stability when sitting (Flatters et al. 2014). Nonetheless, as the children could decide to match the exact target movement or take the shortest possible distance to keep up with the target, other age-related skills such as sequential neural computation (Lookadoo et al. 2017) could relate to the reduced performance in children with NDDs (Hyde & Wilson 2011).

Our findings of significant linear relationships between neurodevelopmental symptom load and tracking difficulties highlight the importance of identifying motor problems in this population. The relationship stayed significant even when removing the item related to motor function from the ESSENCE-Q score (*n* = 5, 8.6%, who marked considerations [“Yes” or “Maybe/A little”]). Problems related to attention (*n* = 15, 25.9%), or hyperactivity (*n* = 18, 31.0%), which can also influence inhibitory control and movement correction were more often reported. The questionnaire identified a subgroup of children who may have a neurodevelopmental disorder. The estimated rate of children with a potential NDD was 29.3–38.0%, which may be interpreted as high compared to the expected NDD prevalence of approximately 10% (Gillberg 2010). However, this is not surprising, as some of our participants were actively recruited through the neuropsychiatry clinic and research clinic (*n* = 9), leading to a higher frequency of neurodevelopmental problems than in the general population, and a more widespread distribution for analysis. In the children recruited at preschools the estimated rate was 22.4% (score ≥ 4) or 30.6% (score ≥ 3).

Here we used the ESSENCE-Q scores as a continuous variable. Our findings inform about the presence and load of neurodevelopmental symptoms at a group level and therefore we cannot make conclusions based on specific diagnoses. Nonetheless, our findings may be important for understanding motor difficulties and development in children with different neurodevelopmental symptoms and how these issues influence specific strategies in visuomotor tracking.

### Overshooting

Our findings that longitudinal adjustments influenced tracking performance support the idea of a forward model for predictive control during movement (Mathew & Cook 1990; Shadmehr et al. 2010; Stapel et al. 2015). We identified an overshooting mechanism that reduced tracking performance in children with neurodevelopmental symptoms, who tended to be more outside the target area. In this aspect, we define “overshooting” as when their positioning of digit touch is too much forward of the target, reducing performance. A medium effect size was observed (*η*^2^ = 0.074), which indicates that this finding has practical application and can have important implications for understanding children’s motor behaviour in the context of NDDs. By considering the idea that motor prediction is a dominant factor for motor control (López-Moliner et al. 2019), our findings may be a sign of specific reduced flexibility in NDDs. Prediction requires the inclusion of new external information together with the reduction of the weight of the previous motor prediction and issues with either of these functions may lead to overshooting the target. Overshooting could be explained by an overestimation of the distance to the target, as can occur in dysmetria (Glazebrook et al. 2008; Gowen & Miall 2005; Katschmarsky et al. 2001; Nazarali et al. 2009). However, considering previous findings of an increased reactivity for unexpected events in adults with NDDs (Marzinzik et al. 2012; Neely et al. 2017), deficits in inhibitory control (Gilbert et al. 2019; Macneil et al. 2011; Mostofsky et al. 2006; Schmitt et al. 2018; Valori et al. 2022), and reduced utilization of motor synergies required for abrupt alternation, a difficulty with predictive control seems to be more plausible explanation (Cannon et al. 2021; Emanuele et al. 2021, 2022; Oliveira et al. 2006). Nonetheless, the trajectories followed dynamic motion profiles based on a power-law relationship between curvature and speed, thus they were, specifically targeting delicate and online motion prediction. It is, however, important to note that we did not analyse specific parts of the trajectories, but the whole trajectories. Additionally, we did not assess children's posture, although children's over-adjustment could depend on how far their touch reached away from the body. The zigzag trajectories were lightly tilted, away from the body, meaning that we did not investigate when sliding towards the body.

The negative relationship between the longitudinal position in the zigzag tracking was related to reduced performance in children without NDD symptoms, which can be interpreted as a more general reduction in processing speed needed to include new information into the predictive model.

Our current results shed new light on a potential controlling mechanism for visuomotor tracking, revealing motor deficits in children with neurodevelopmental symptoms that lead to potential over-adjustments in the target movement direction.

### Error correction

Our findings further indicate that the reduced correction for lateral deviations in the continuous tracking may relate to specific problems with smooth directional control in NDDs (Rodger et al. 2003; Scharoun et al. 2013). Note, however, that the relationship between neurodevelopmental symptoms and performance in the tracking of continuous trajectory was not found to be significant (*p* = 0.090) when including the interaction term between ESSENCE-Q score and lateral variability, suggesting overall reduced performance in children with NDD symptoms. Participants who exhibited neurodevelopmental symptoms demonstrated specific challenges in making corrections. Taking into account that prediction plays a significant role in motor control (López-Moliner et al. 2019), our findings suggest that neurodevelopmental symptoms reduce the awareness and the ability to correct for variability within a predictive model. These difficulties in correction could potentially imply a decrease in adaptiveness and self-monitoring, as has been previously noted (Kagerer et al. 2004; Kurdziel et al. 2015; Pomè et al. 2023; Shiels & Hawk 2010). It is also likely that reduced awareness of motor variability could stem from sensory challenges (Ghanizadeh 2011; Tran et al. 2022; Whyatt & Craig 2013). Here, however, only a small effect size (*η*^2^ = 0.023) was observed in the interaction between ESSENCE-Q score and lateral variability, meaning that practical applicability may be limited.

Prediction errors have been predominantly investigated with delayed feedback, such as in trial-to-trial corrections (López-Moliner et al. 2019), or without including dynamic visual feedback in tracking (Tirosh et al. 2006). To enact effective real-time corrections, we argue that visual feedback is important, in particular when studying specific motor mechanisms in children with NDDs, as this has been found to improve motor performance (Liu & Breslin 2013; Yang et al. 2014). Lack of dynamic visual feedback may have a role in why some more specific features for directional control have been found non-significant to NDDs in other studies, e.g., Tirosh et al. (2006).

Importantly, we acknowledge that variability does not have to be seen as a negative feature of motor control, as it can indicate flexibility or adaptation (Latash 2012). We found a significant negative relationship between lateral variability and tracking performance, confirming that the variability overall had a negative influence on performance—yet we cannot rule out that a portion of the variability could have been positive for tracking performance, as regulations are needed for directional control. Another explanation for our findings could be that the relative proportion of negative variability increases with neurodevelopmental symptom load. However, here the variability was assumed to be detrimental to performance, based on the fact that continuous tracking requires smooth coordination (Selen et al. 2006). Our results provide additional information about underlying motor adaptation, revealing reduced correction for lateral variability in children with neurodevelopmental symptoms.

### Limitations and strengths

This study comes with some limitations and considerations. First, the sample size in this study was relatively small (*n* = 58), and therefore, generalizability may be limited. Note that we took specific steps to diversify the population, by collecting data in different schools in various neighbourhoods.

Second, the use of tablets for data collection introduced a potential confounding variable. While tablet usage has been adopted across different preschools/schools, variations in the familiarity of the interface of the tablet might have influenced their responses and behaviour. Collecting data related to previous tablet experience could enhance the analysis process collection for future studies.

The ESSENCE-Q questionnaire used to gather information relies on parent ratings. Parent-reported data can be influenced by subjective perceptions and biases, potentially affecting the accuracy of the reported information. Combining parent reports with other sources of data, such as teacher assessments or direct observations, could likely provide a more comprehensive and reliable picture of the participants' characteristics. However, it is important to note that the ESSENCE-Q instructs parents to indicate concerns coming from either themselves or others. Consequently, the ESSENCE-Q has the potential to encompass concerns from other than the parents themselves.

This tablet-based motor test has previously only been tested in children with a specific neuropsychiatric syndrome [PANS] (Thorsson et al. 2023). This previous study showed strong correlations between this test and another motor test [The Beery-Buktenica Developmental Test of Visual-Motor Integration 6th Edition assessment, Motor Coordination subtest] (Thorsson et al. 2023), but the psychometric properties are not yet fully known.

We excluded data from children who either did not engage with the task or had difficulties understanding the more complex trajectories. An alternative approach could be to include trials that were completed to less than 80%, but we estimate that this could reduce the consistency of the final data.

Future studies including a larger number of children may allow the unveiling of even more specific mechanisms for regulation and correction of longitudinal and lateral errors in visuomotor tracking, and thereby get an even more comprehensive picture of motor control in NDDs.

### Implications

The ability to regulate and correct directional features of timed movement is essential in daily life such as in self-care (e.g., brushing teeth or showering) and certain types of communication (e.g., writing or drawing). These are movements that have been previously identified as being particularly difficult for individuals with NDDs (Anzulewicz et al. 2016; Farmer et al. 2016; Prunty & Barnett 2019; Willoughby & Polatajko 1995).

The current study suggests that neurodevelopmental symptoms are associated with difficulties in regulating timed movement of alternating directions. This was observed in a specific visuomotor tracking task previously used specifically in children with a condition specifically affecting motor control (PANS). The children's reduced tracking performance was driven by over-adjustments in the target movement direction. Conversely, in children without NDD symptoms, reduced performance was more driven by not being behind the target. Additionally, children with NDD-related symptoms were less flexible in correcting for lateral deviations in the tracking of continuous direction. Finally, our findings highlight that age is an important predictor of motor performance in the tracking of both continuous and alternating directions.

In conclusion, our findings confirm that deficits in online correction relate to general NDD symptoms, highlighting the importance of considering motor problems when a child shows NDD symptoms. This new tool may provide possibilities for better identification and understanding of problems even before a specific diagnosis is given in children with NDDs.

### Supplementary Information

Below is the link to the electronic Supplementary material.

Supplementary file1 (PDF 1318 KB)

## Data Availability

The study reported in this article was not pre-registered. The data have not been made available on a permanent third-party archive because participants were not asked to consent for their data to be made publicly available, even anonymized. Data are available upon request from those who wish to collaborate with us, via a visitor agreement with the University of Gothenburg, if appropriate, under existing ethics approval.
