# Supplementary material for: Visuomotor tracking strategies in children: associations with neurodevelopmental symptoms
Source: Exp Brain Res. 2023 Dec 11;242(2):337–53. doi: 10.1007/s00221-023-06752-0 (PMC11297076; doi:10.1007/s00221-023-06752-0)
Supplement: Supplementary file 1 — Supplementary file1 (PDF 1318 KB) [file 221_2023_6752_MOESM1_ESM.pdf]

Experimental Brain Research

SUPPLEMENTARY MATERIAL

Supplementary material

Visuomotor tracking strategies in children: associations with neurodevelopmental symptoms

Max Thorsson[1✉], Martyna A. Galazka[1, 2], Mats Johnson[1], Jakob Åsberg Johnels[1,3], and Nouchine Hadjikhani[1,4]

[1] Gillberg Neuropsychiatry Centre, Institute of Neuroscience and Physiology, Sahlgrenska Academy, University of Gothenburg, Gothenburg, Sweden
[2] Division of Cognition and Communication, Department of Applied Information Technology, University of Gothenburg, Gothenburg, Sweden
[3] Section of Speech and Language Pathology, Institute of Neuroscience and Physiology, Sahlgrenska Academy, University of Gothenburg, Gothenburg, Sweden
[4] Athinoula A. Martinos Center for Biomedical Imaging, Harvard Medical School, Massachusetts General Hospital, Boston, MA, USA

✉ Corresponding author (max.thorsson@gu.se)

Supplementary content

Within this document, you will find a collection of supplementary tables methods, and testing, each complementing and expanding upon the content featured in the main article. In the main article, you will discover in-depth explanations for the variables used, and research questions (RQs), while this document serves to provide additional context and details through supplementary tables, figures, and methods. Note that images of trajectories are the content of the motor test and are therefore subject to copyright.

Tables include the dependent (Dep.) variable, the ordinary least squares (OLS) models' total and adjusted (Adj.) R^2, F-statistic, significance, covariance type, number of (No.) observations, degrees of freedom (Df), intercept, predictors; and their coefficients' (coef), standard errors (std err), t-statistics (t), and significance ($p>|t|$), as well as the boundary values of the 95% confidence interval ([0.025, 0.975]). For interpretation, please note that the performance, longitudinal and lateral features have been standardized. Furthermore, other variables have been mean-centred. In the following tables and figures, the total ESSENCE-Q score is denoted "ESSENCE". Furthermore, we display partial regression plots, obtained using Statsmodels, v. 0.14.0 (Seabold & Perktold, 2010), which represent the relationship between an independent and dependent variable while isolating the impact of all other independent variables (Olive et al., 2015). Note, that the x- and y -axes are not the actual variables. In other words, these plots help us examine how independent and dependent variables relate when we account for other variables or remove the effects of other factors. Note that heteroscedasticity robust covariance estimators (HC3) were used to calculate standard errors.

Supplementary tables and figures

Results and partial regressions in *Tables 1 – 6*, as well as *Figures 1– 6*, are based on the methods described in the main article.

RQ1: Longitudinal error

Table 1 Regression results for RQ1A

**Dep. Variable:**	LongitudinalError (zigzag)			**R-squared:**		0.401
**Model:**	OLS			**Adj. R-squared:**		0.368
**No. Observations:**	58			**F-statistic:**		9.105
**Df Residuals:**	54			**Prob (F-statistic):**		<0.001
**Df Model:**	3			**Covariance Type:**		HC3
	**coef**	**std err**	***t***	***p*>|*t*|**	**[0.025**	**0.975]**
**Intercept**	-0.2833	0.127	-2.234	0.030	-0.538	-0.029
**Age**	-0.3718	0.314	-1.183	0.242	-1.002	0.258
**Age^2**	0.0049	0.021	0.231	0.818	-0.038	0.047
**ESSENCE**	0.0950	0.037	2.588	0.012	0.021	0.169

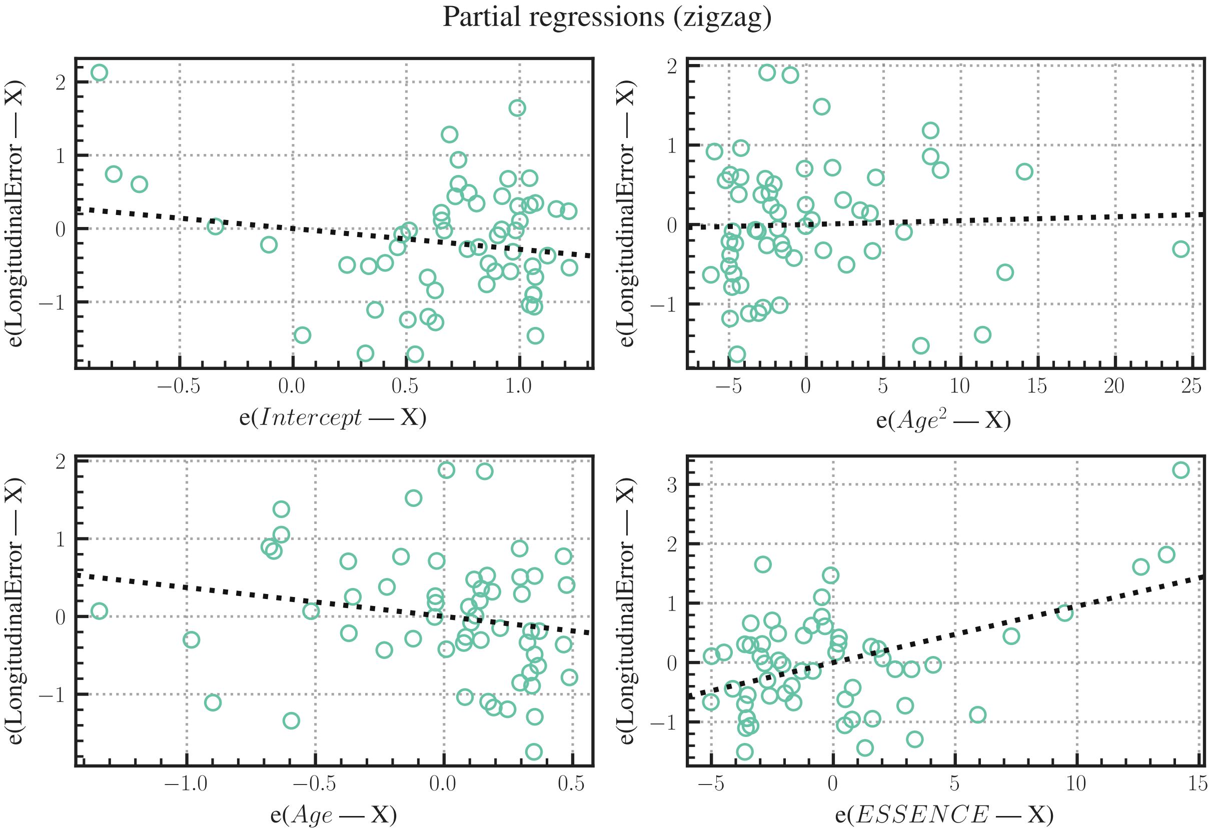

Fig. 1 Partial regression plots that show the relationship between independent and dependent variables while considering the influence of adding other independent variables

Table 2 Regression results for RQ1B

**Dep. Variable:**	LongitudinalError (spiral)			**R-squared:**		0.592
**Model:**	OLS			**Adj. R-squared:**		0.568
**No. Observations:**	50			**F-statistic:**		21.01
**Df Residuals:**	3			**Prob (F-statistic):**		<0.001
**Df Model:**	54			**Covariance Type:**		HC3
	**coef**	**std err**	***t***	***p*>|*t*|**	**[0.025**	**0.975]**
**Intercept**	-0.1272	0.108	-1.175	0.246	-0.345	0.090
**Age²**	0.0498	0.021	2.345	0.023	0.007	0.092
**Age**	-1.1296	0.318	-3.555	0.001	-1.768	-0.491
**ESSENCE**	0.0416	0.027	1.530	0.132	-0.013	0.096

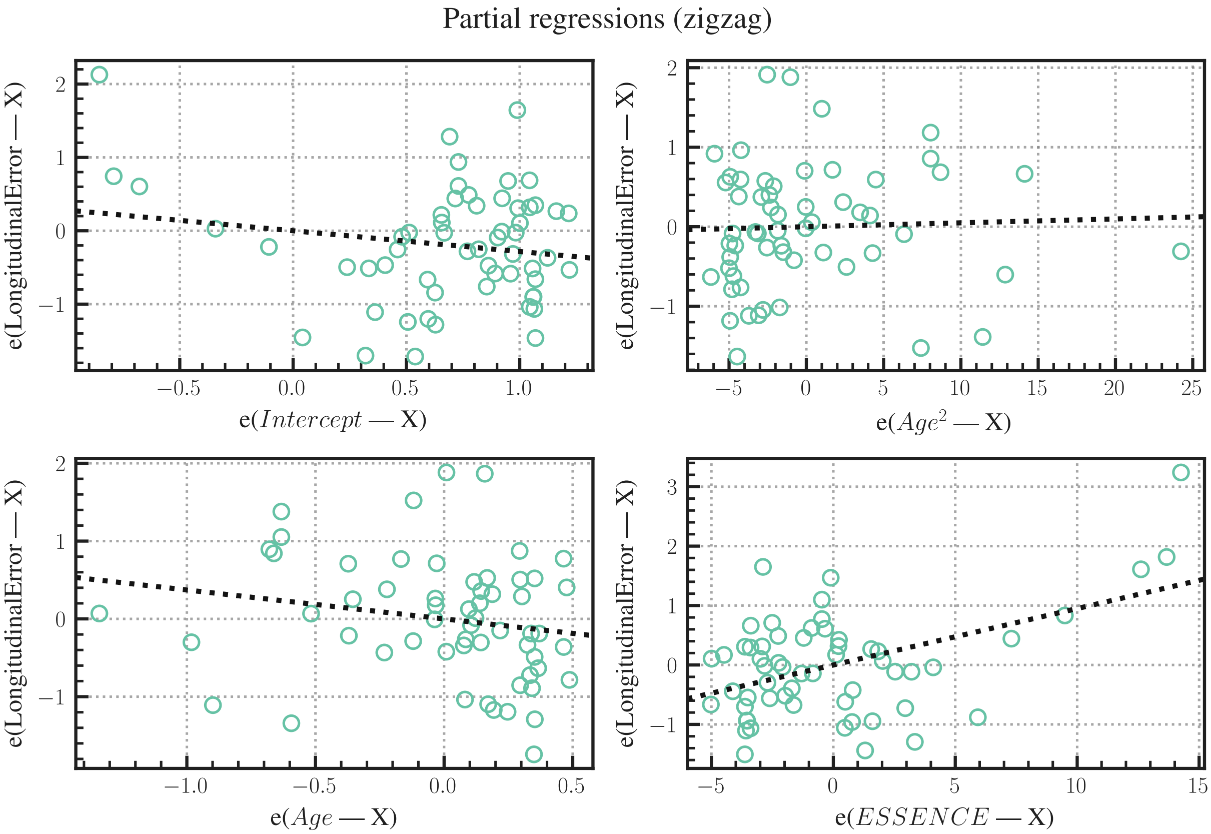

Fig. 2 Partial regression plots that show the relationship between independent and dependent variables while considering the influence of adding other independent variables

RQ2: Tracking performance and longitudinal regulation in zigzag tracking

Table 3 Regression results for RQ2A

**Dep. Variable:**	Performance (zigzag)	**R-squared:**	0.433
**Model:**	OLS	**Adj. R-squared:**	0.401
**No. Observations:**	58	**F-statistic:**	22.10
**Df Residuals:**	54	**Prob (F-statistic):**	<0.001
**Df Model:**	3	**Covariance Type:**	HC3

	**coef**	**std err**	***t***	***p*>|*t*|**	**[0.025**	**0.975]**
**Intercept**	-0.1152	0.126	-0.911	0.366	-0.369	0.138
**Age**	0.5513	0.265	2.078	0.043	0.019	1.083
**Age^2**	-0.0146	0.017	-0.880	0.383	-0.048	0.019
**ESSENCE**	-0.0613	0.022	-2.754	0.008	-0.106	-0.017

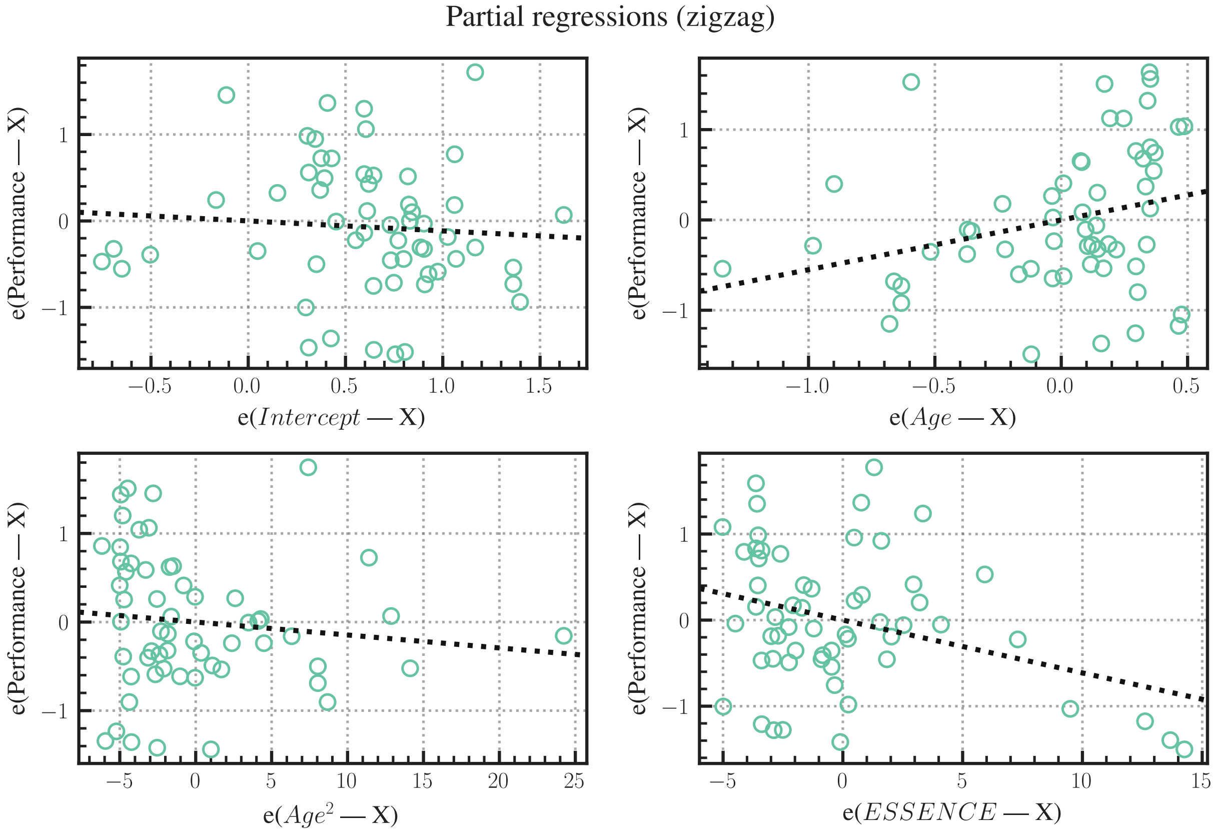

Fig. 3 Partial regression plots that show the relationship between independent and dependent variables while considering the influence of adding other independent variables

Table 4 Regression results for RQ2B

**Dep. Variable:**	Performance (zigzag)	**R-squared:**	0.548
**Model:**	OLS	**Adj. R-squared:**	0.504
**No. Observations:**	58	**F-statistic:**	18.30
**Df Residuals:**	52	**Prob (F-statistic):**	<0.001
**Df Model:**	5	**Covariance Type:**	HC3

	**coef**	**std err**	***t***	***p*>|*t*|**	**[0.025**	**0.975]**
**Intercept**	-0.2565	0.121	-2.115	0.039	-0.500	-0.013
**Age**	0.7184	0.251	2.861	0.006	0.215	1.222
**Age^2**	-0.0268	0.016	-1.666	0.102	-0.059	0.005
**LongitudinalPosition**	0.3848	0.117	3.295	0.002	0.150	0.619
**ESSENCE**	-0.0475	0.030	-1.568	0.123	-0.108	0.013
**LongitudinalPosition:ESSENCE**	-0.0401	0.013	-3.047	0.004	-0.066	-0.014

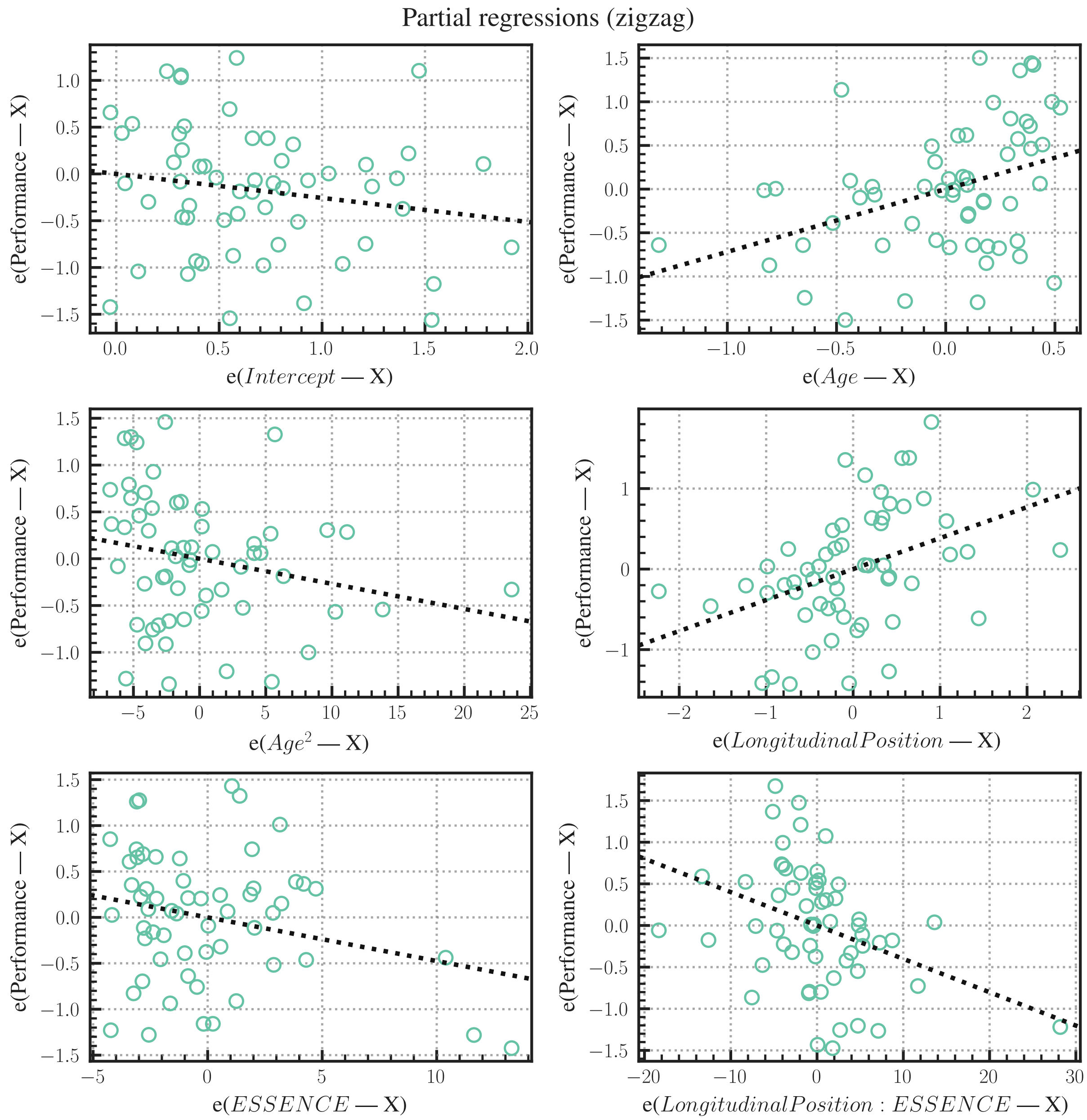

Fig. 4 Partial regression plots show the relationship between variables while considering added independent variables

RQ3: Tracking performance and perpendicular adaptiveness in spiral tracking

Table 5 Regression results for RQ1A

**Dep. Variable:**	Performance (spiral)	**R-squared:**	0.615
**Model:**	OLS	**Adj. R-squared:**	0.592
**No. Observations:**	54	**F-statistic:**	27.04
**Df Residuals:**	50	**Prob (F-statistic):**	<0.001
**Df Model:**	3	**Covariance Type:**	HC3

	**coef**	**std err**	***t***	***p*>|*t*|**	**[0.025**	**0.975]**
**Intercept**	-0.5963	0.140	-4.248	<0.001	-0.878	-0.314
**Age**	1.2519	0.295	4.243	<0.001	0.659	1.844
**Age^2**	-0.0583	0.020	-2.952	0.005	-0.098	-0.019
**ESSENCE**	-0.0376	0.030	-1.256	0.215	-0.098	0.023

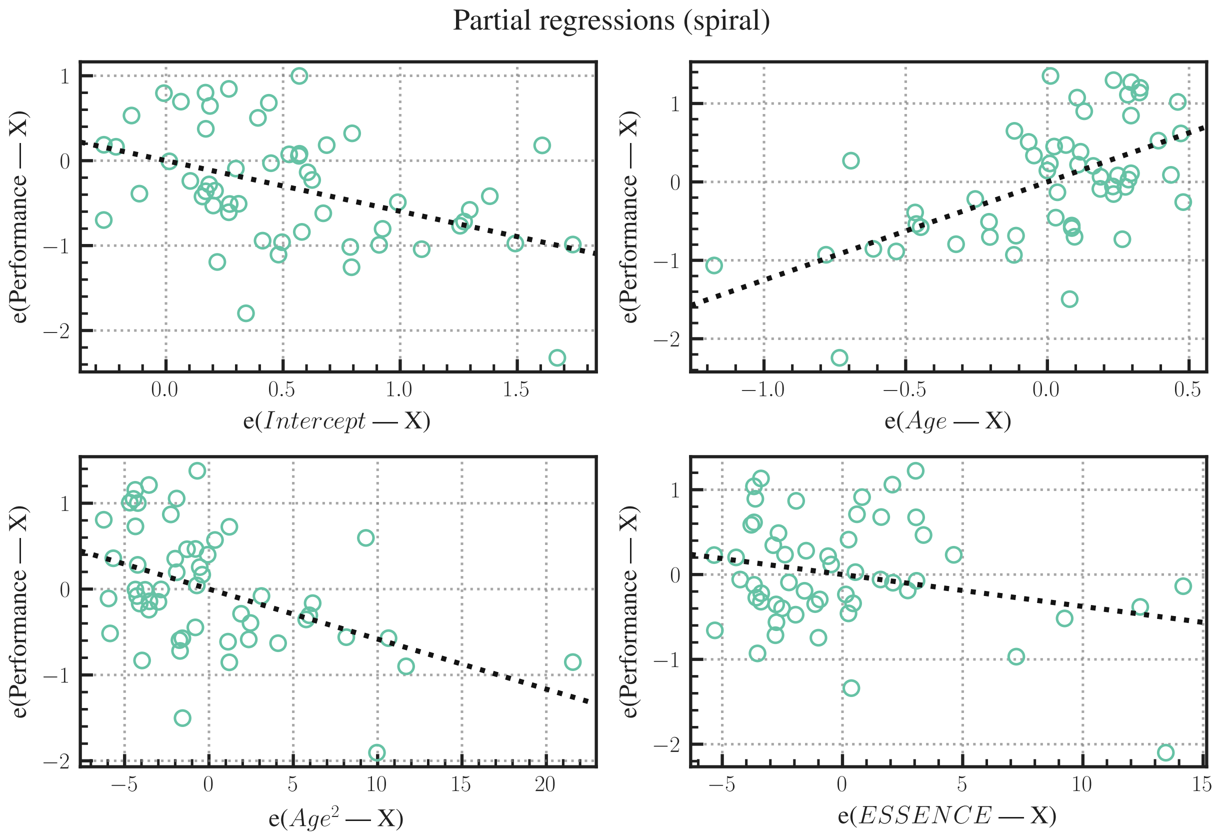

Fig. 5 Partial regression plots that show the relationship between independent and dependent variables while considering the influence of adding other independent variables

Table 6 Regression results for RQ3B

**Dep. Variable:**	Performance (spiral)	**R-squared:**	0.826
**Model:**	OLS	**Adj. R-squared:**	0.808
**No. Observations:**	54	**F-statistic:**	109.9
**Df Residuals:**	48	**Prob (F-statistic):**	<0.001
**Df Model:**	5	**Covariance Type:**	HC3

	**coef**	**std err**	t	$p>\|t\|$	**[0.025**	**0.975]**
**Intercept**	-0.2360	0.116	-2.032	0.048	-0.470	-0.002
**Age**	0.3818	0.198	1.925	0.060	-0.017	0.781
**Age^2**	-0.0138	0.012	-1.192	0.239	-0.037	0.009
**LateralVariability**	-0.6756	0.074	-9.160	<0.001	-0.824	-0.527
**ESSENCE**	-0.0179	0.010	-1.732	0.090	-0.039	0.003
**LateralVariability:ESSENCE**	-0.0451	0.011	-4.014	<0.001	-0.068	-0.023

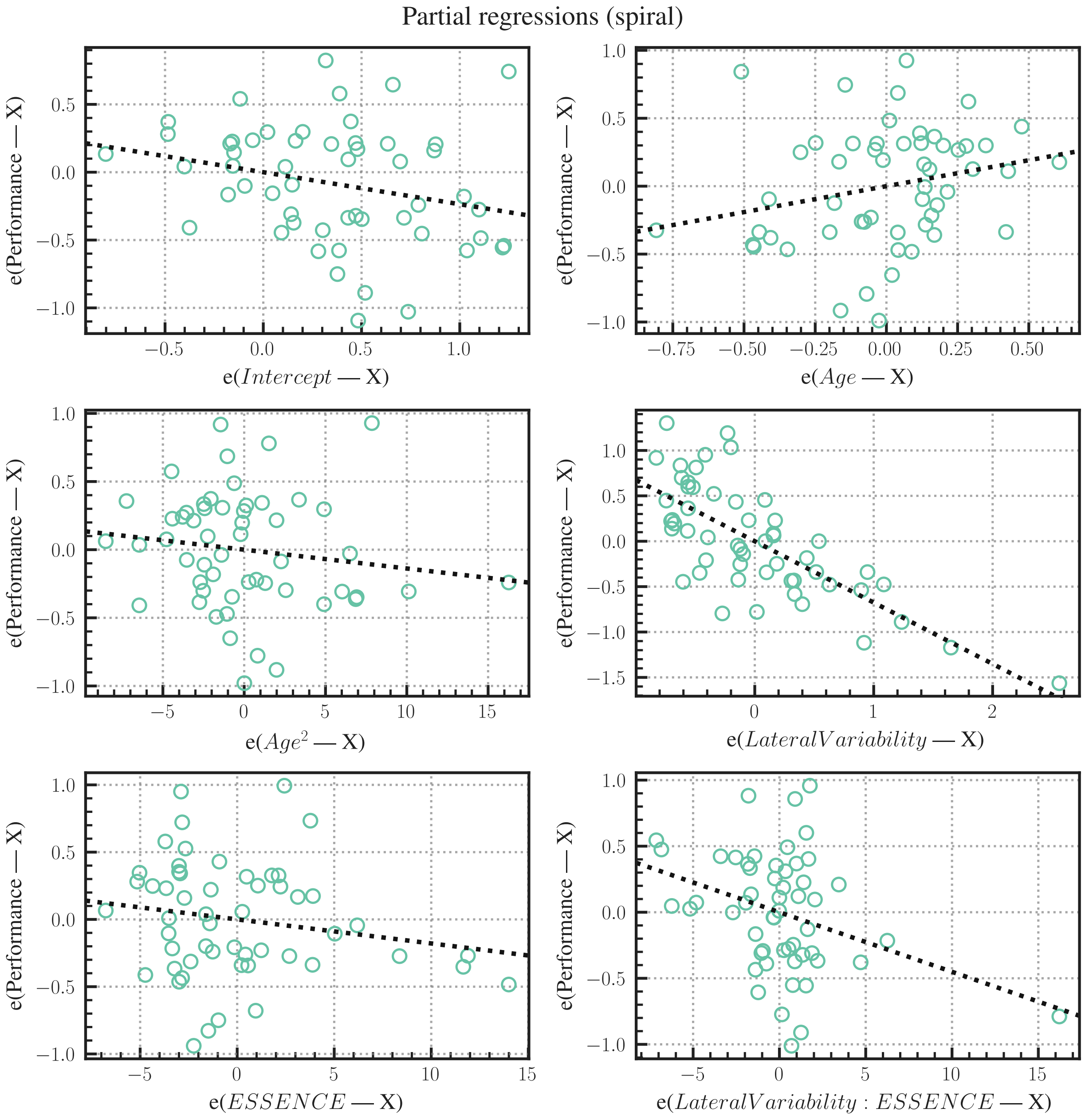

Fig. 6 Partial regression plots show the relationship between variables while considering added independent variables

Supplementary testing (without motor item)

ESSENCE-Q has a question concerning motor development, which inherently creates the potential for a correlation between the total score and motor control. In order to rule out potential ambiguity, we conducted statistical tests while excluding the scores from the motor item. See the results when excluding the motor item in *Tables 7 – 12*.

RQ1: Longitudinal error

Table 7 Regression results for RQ1A, without the motor development item on ESSENCE-Q

**Dep. Variable:**	LongitudinalError (zigzag)	**R-squared:**	0.385
**Model:**	OLS	**Adj. R-squared:**	0.351
**No. Observations:**	58	**F-statistic:**	8.802
**Df Residuals:**	54	**Prob (F-statistic):**	<0.001
**Df Model:**	3	**Covariance Type:**	HC3

	**coef**	**std err**	***t***	***p*>|*t*|**	**[0.025**	**0.975]**
**Intercept**	-0.2753	0.127	-2.171	0.034	-0.529	-0.021
**Age**	-0.3552	0.315	-1.127	0.265	-0.987	0.277
**Age^2**	0.0037	0.021	0.176	0.861	-0.039	0.046
**ESSENCE**	0.0968	0.040	2.434	0.018	0.017	0.176

Table 8 Regression results for RQ1B, without the motor development item on ESSENCE-Q

**Dep. Variable:**	LongitudinalError (spiral)	**R-squared:**	0.588
**Model:**	OLS	**Adj. R-squared:**	0.563
**No. Observations:**	54	**F-statistic:**	20.76
**Df Residuals:**	50	**Prob (F-statistic):**	<0.001
**Df Model:**	3	**Covariance Type:**	HC3

	**coef**	**std err**	***t***	***p*>|*t*|**	**[0.025**	**0.975]**
**Intercept**	-0.1218	0.108	-1.127	0.265	-0.339	0.095
**Age**	-1.1206	0.318	-3.521	0.001	-1.760	-0.481
**Age^2**	0.0492	0.021	2.315	0.025	0.007	0.092
**ESSENCE**	0.0419	0.029	1.446	0.154	-0.016	0.100

RQ2: Tracking performance and longitudinal regulation in zigzag tracking

Table 9 Regression results for RQ2A, without the motor development item on ESSENCE-Q

**Dep. Variable:**	Performance (zigzag)	**R-squared:**	0.425
**Model:**	OLS	**Adj. R-squared:**	0.393
**No. Observations:**	58	**F-statistic:**	21.71
**Df Residuals:**	54	**Prob (F-statistic):**	<0.001
**Df Model:**	3	**Covariance Type:**	HC3

	**coef**	**std err**	***t***	***p*>|*t*|**	**[0.025**	**0.975]**
**Intercept**	-0.1188	0.127	-0.936	0.353	-0.373	0.136
**Age**	0.5396	0.266	2.032	0.047	0.007	1.072
**Age^2**	-0.0138	0.017	-0.833	0.409	-0.047	0.019
**ESSENCE**	-0.0620	0.025	-2.463	0.017	-0.112	-0.012

Table 10 Regression results for RQ2B, without the motor development item on ESSENCE-Q

**Dep. Variable:**	Performance (zigzag)			**R-squared:**		0.541
**Model:**	OLS			**Adj. R-squared:**		0.497
**No. Observations:**	58			**F-statistic:**		16.71
**Df Residuals:**	52			**Prob (F-statistic):**		<0.001
**Df Model:**	5			**Covariance Type:**		HC3
	**coef**	**std err**	**t**	***p*>|t|**	**[0.025**	**0.975]**
**Intercept**	-0.2526	0.122	-2.065	0.044	-0.498	-0.007
**Age**	0.7067	0.253	2.793	0.007	0.199	1.214
**Age^2**	-0.0260	0.016	-1.606	0.114	-0.059	0.006
**LongitudinalPosition**	0.3822	0.118	3.236	0.002	0.145	0.619
**ESSENCE**	-0.0456	0.032	-1.413	0.163	-0.110	0.019
**LongitudinalPosition:ESSENCE**	-0.0450	0.015	-2.952	0.005	-0.076	-0.014

RQ3: Tracking performance and perpendicular adaptiveness in spiral tracking

Table 11 Regression results for RQ3A, without the motor development item on ESSENCE-Q

**Dep. Variable:**	Performance (zigzag)			**R-squared:**		0.611
**Model:**	OLS			**Adj. R-squared:**		0.588
**No. Observations:**	54			**F-statistic:**		26.90
**Df Residuals:**	50			**Prob (F-statistic):**		<0.001
**Df Model:**	3			**Covariance Type:**		HC3
	**coef**	**std err**	***t***	***p*>|*t*|**	**[0.025**	**0.975]**
**Intercept**	-5.0245	1.007	-4.991	<0.001	-7.047	-3.002
**Age**	1.2436	0.296	4.206	<0.001	0.650	1.837
**Age^2**	-0.0577	0.020	-2.921	0.005	-0.097	-0.018
**ESSENCE**	-0.0378	0.031	-1.220	0.228	-0.100	0.024

Table 12 Regression results for RQ3B, without the motor development item on ESSENCE-Q

**Dep. Variable:**	Performance (spiral)			**R-squared:**		0.826
**Model:**	OLS			**Adj. R-squared:**		0.808
**No. Observations:**	54			**F-statistic:**		98.38
**Df Residuals:**	48			**Prob (F-statistic):**		<0.001
**Df Model:**	5			**Covariance Type:**		HC3
	**coef**	**std err**	***t***	***p*>|*t*|**	**[0.025**	**0.975]**
**Intercept**	0.2364	0.118	2.008	0.050	-0.473	<0.001
**Age**	0.3762	0.201	1.868	0.068	-0.029	0.781
**Age^2**	0.0135	0.012	1.154	0.254	-0.037	0.010
**LateralVariability**	0.6800	0.075	9.008	<0.001	-0.832	-0.528
**ESSENCE**	0.0188	0.012	1.621	0.111	-0.042	0.005
**LateralVariability:ESSENCE**	0.0486	0.014	3.494	0.001	-0.077	-0.021

Supplementary testing (zigzag)

The following analysis was conducted to rule out potential ambiguity that could occur when measuring the participant's touch position on the longitudinal axis, relative to the target position and direction. This type of calculation comes with a risk that if the participants lag behind a sharp turn, their position will be influenced by changes in the target's movement direction, and could thereby be misinterpreted as overshooting while lagging behind the target. To clarify that this was not the case, the zigzags were segmented by lines orthogonal to the tangential angle at the peaks of the zigzag turns, so that the case of lagging behind the target would not show as overshooting, see *Fig. 7*.

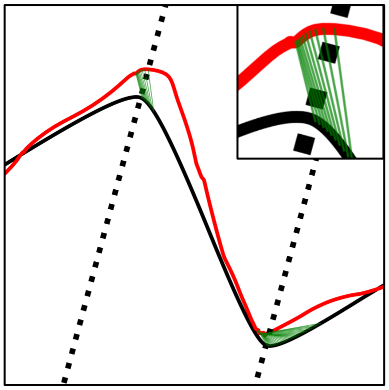

Fig. 7 Zigzags were separated into segments (the dotted lines separate the zigzag). The green lines between the participant's trajectory (red line) and the target trajectory (solid line) represent the points that were in a previous segment while the target was in the next. A magnification is shown in the upper right corner. The data is from an example participant

If the participant touch coordinates had not entered the new segment (that the target was in), they were instead rotated relative tangential angle of the peak, which was the same as the average direction of the zigzags. We found only minimal changes in coefficients and *p*-values, confirming that the potential effect, that lagging behind in abrupt turn would indicate overshooting, did not influence our analysis. Results for the regressions are shown in *Tables 13* and *14*. As a final control analysis, we also included the case if the participant was in a segment before the target position, in the aforementioned analysis, neither did this change the results, to more than minimal changes in coefficients and *p*-values, further corroborating the interpretation of longitudinal position as a metric for motor regulation.

RQ1: Longitudinal error

Table 13 Regression results for RQ1A when estimating longitudinal error for segmented by turns

**Dep. Variable:**	LongitudinalError (zigzag)	**R-squared:**	0.346
**Model:**	OLS	**Adj. R-squared:**	0.309
**No. Observations:**	58	**F-statistic:**	9.775
**Df Residuals:**	54	**Prob (F-statistic):**	<0.001
**Df Model:**	3	**Covariance Type:**	HC3

	**coef**	**std err**	***t***	***p*>|*t*|**	**[0.025**	**0.975]**
**Intercept**	-0.2396	0.133	-1.800	0.078	-0.506	0.027
**Age**	-0.3205	0.312	-1.028	0.309	-0.946	0.305
**Age^2**	0.0022	0.020	0.107	0.915	-0.039	0.043
**ESSENCE**	0.0803	0.035	2.314	0.024	0.011	0.150

RQ2: Tracking performance and longitudinal regulation in zigzag tracking

Table 14 Regression results for RQ2B when estimating longitudinal position segmented by turns

**Dep. Variable:**	Performance (zigzag)			**R-squared:**		0.579
**Model:**	OLS			**Adj. R-squared:**		0.539
**No. Observations:**	58			**F-statistic:**		18.18
**Df Residuals:**	52			**Prob (F-statistic):**		<0.001
**Df Model:**	5			**Covariance Type:**		HC3
	**coef**	**std err**	***t***	***p*>|*t*|**	**[0.025**	**0.975]**
**Intercept**	-0.2502	0.123	-2.036	0.047	-0.497	-0.004
**Age**	0.7194	0.251	2.865	0.006	0.215	1.223
**Age^2**	-0.0273	0.016	-1.675	0.100	-0.060	0.005
**LongitudinalPosition**	0.4305	0.122	3.537	0.001	0.186	0.675
**ESSENCE**	-0.0492	0.030	-1.642	0.107	-0.109	0.011
**LongitudinalPosition:ESSENCE**	-0.0427	0.014	-3.043	0.004	-0.071	-0.015

Supplementary testing (spiral)

The following analysis was conducted to rule out potential ambiguity that could occur when measuring the variations in lateral position (lateral variability) relative to the target movement position and direction. In order to confirm that the lateral variability was not caused by the participant simply lagging behind the target while following the path, we estimated the lateral variability relative to the closest position on the path, instead of to the target position, see *Fig. 8*. To avoid the closest position from being on another loop of the spiral, we sat a limit of 90 degrees away from the target movement direction, behind the target of the points at which the position was identified. In this supplementary analysis, we found that only minimal changes occurred in the decimals of the coefficients and *p*-values of the regression. If the participants had been delayed and perfectly followed the trajectory, this regression would reasonably not be significant to tracking performance or in the interaction with ESSENCE-Q score. Results for the regressions are shown in *Table 15*. As a final control, we also considered the case if the participant was following the path in front of the target position, thereby creating variability; by shifting the 90-degree-threshold, 45 degrees in the direction of the target movement. By shifting the inclusion range, we also included potential distances to the path, in front of the target. These changes did not alter the results, to more than minimal changes in coefficients and p-values.

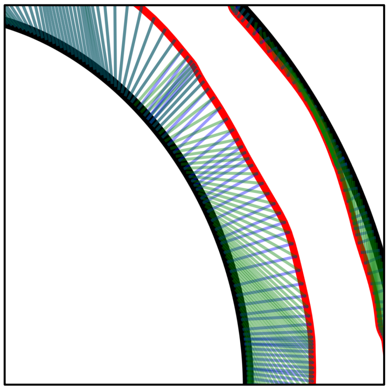

Fig. 8 The lateral position can be measured relative to the target position (green lines) and relative to the closest position on the target path. The participant's trajectory (red line) and the target trajectory (solid black line) are from one example participant

RQ3: Tracking performance and perpendicular adaptiveness in spiral tracking

Table 15 Regression results for RQ3B when estimating variability relative to the path

**Dep. Variable:**	Performance (spiral)	**R-squared:**	0.797
**Model:**	OLS	**Adj. R-squared:**	0.776
**No. Observations:**	54	**F-statistic:**	88.75
**Df Residuals:**	48	**Prob (F-statistic):**	<0.001
**Df Model:**	5	**Covariance Type:**	HC3

	**coef**	**std err**	***t***	***p*>|*t*|**	**[0.025**	**0.975]**
**Intercept**	-0.2891	0.130	-2.227	0.031	-0.550	-0.028
**Age**	0.4988	0.224	2.227	0.031	0.048	0.949
**Age^2**	-0.0195	0.013	-1.504	0.139	-0.046	0.007
**LateralVariability**	-0.6009	0.091	-6.633	<0.001	-0.783	-0.419
**ESSENCE**	-0.0156	0.012	-1.344	0.185	-0.039	0.008
**LateralVariability:ESSENCE**	-0.0484	0.016	-2.983	0.004	-0.081	-0.016

References

Olive, D., Watagoda, L., & Rupasinghe Arachchige Don, H. (2015). Visualizing and Testing the Multivariate Linear Regression Model. *International Journal of Statistics and Probability*, *4*. https://doi.org/10.5539/ijsp.v4n1p126

Seabold, S., & Perktold, J. (2010). Statsmodels: Econometric and Statistical Modeling with Python. Proceedings of the 9th Python in Science Conference,
